# Design and Evaluation of a Low-Power Wide-Area Network (LPWAN)-Based Emergency Response System for Individuals with Special Needs in Smart Buildings

**DOI:** 10.3390/s24113433

**Published:** 2024-05-26

**Authors:** Habibullah Safi, Ali Imran Jehangiri, Zulfiqar Ahmad, Mohammed Alaa Ala’anzy, Omar Imhemed Alramli, Abdulmohsen Algarni

**Affiliations:** 1 Department of Computer Science and Information Technology, Hazara University, Mansehra 21300, Pakistan; habibullah.hairan@gmail.com (H.S.); ali_imran@hu.edu.pk (A.I.J.); 2 Department of Computer Science, SDU University, Almaty 040900, Kazakhstan; 3 Department of Networks and Communications, Faculty of Information Technology, Misurata University, Misurata P.O. Box 2478, Libya; omar.alramli@it.misuratau.edu.ly; 4 Department of Computer Science, King Khalid University, Abha 61421, Saudi Arabia; a.algarni@kku.edu.sa

**Keywords:** emergency response, Heltec, WiFi, LoRa 32, IoT, LPWAN, wireless sensor networks

## Abstract

The Internet of Things (IoT) is a growing network of interconnected devices used in transportation, finance, public services, healthcare, smart cities, surveillance, and agriculture. IoT devices are increasingly integrated into mobile assets like trains, cars, and airplanes. Among the IoT components, wearable sensors are expected to reach three billion by 2050, becoming more common in smart environments like buildings, campuses, and healthcare facilities. A notable IoT application is the smart campus for educational purposes. Timely notifications are essential in critical scenarios. IoT devices gather and relay important information in real time to individuals with special needs via mobile applications and connected devices, aiding health-monitoring and decision-making. Ensuring IoT connectivity with end users requires long-range communication, low power consumption, and cost-effectiveness. The LPWAN is a promising technology for meeting these needs, offering a low cost, long range, and minimal power use. Despite their potential, mobile IoT and LPWANs in healthcare, especially for emergency response systems, have not received adequate research attention. Our study evaluated an LPWAN-based emergency response system for visually impaired individuals on the Hazara University campus in Mansehra, Pakistan. Experiments showed that the LPWAN technology is reliable, with 98% reliability, and suitable for implementing emergency response systems in smart campus environments.

## 1. Introduction

Throughout history, emergency response systems have been significant in mitigating the negative impacts of natural disasters. Environmental emergencies, such as earthquakes, panic situations, theft, and navigation for the visually impaired, can be particularly challenging [1]. Furthermore, obstacles, including oil spills, chemical spills, attacks, pandemics, nuclear disasters, heavy snowfalls, storms, hurricanes, floods, wild or bush fires, and tsunamis, endanger the safety and welfare of the public at large [2]. Typically, emergency medical services, the fire department, police, and community volunteers initially address these incidents [3]. Effective communication between disaster-stricken areas and disaster management offices is critical, and restoring network services immediately following a disaster is essential for facilitating first responder activities [4]. With the advent of distributed technologies such as the IoT, big data, cloud computing, and 5G cellular networks, the internet has undergone a significant evolution. This transformation has led to the development of cost-effective networks with the capacity to cover large areas. One such network is the Long-Range (LoRa) network, which belongs to the family of low-power wide-area networks (LPWANs) [5,6]. These cutting-edge technologies allow for the placement and maintenance of sensors using a low-power wide-area client, with a maximum range of 30 km in sensor network operational areas [7]. This capability could enable the monitoring of almost all habitable regions without requiring additional management systems. In other words, this can be achieved through device self-communication, such as device-to-device (D2D) and machine-to-machine (M2M) communication technologies. If a mobile device is outside the range of a cellular network, it can communicate using D2D communication with a peer-to-peer wireless communications network. In an emergency, if there is a network connection between the mobile device and the network element, the device sends a message to the network element. If there is no network connection, the device transmits the data to a second mobile device, which connects to a third mobile device using D2D communication [8,9,10]. In an emergency, time is critical and reliability is paramount. Therefore, it is essential to establish an emergency action plan that ensures the prompt provision of rescue resources. To address the challenges posed by such scenarios, we conducted research into an LPWAN-based emergency response system for people with special needs on smart campuses. The experimental results indicate that LPWAN technology is an effective solution for the implementation of emergency response systems on smart campuses.

LPWAN technology has gained significant attention in scientific communities and industry because of its low power consumption, low cost, and long-range communication capabilities, allowing for reliable communication up to 30 km [7,11]. These features make LPWAN ideal for IoT-based wireless sensor networks (WSNs) that transmit minimal data at low rates over long distances with a radio chipset cost of less than USD 2 and an operating cost of USD 1 per device per year [8,9]. LPWAN technology encompasses various low-powered networks that come in different forms and sizes. The increasing demand for IoT in fields such as emergency response systems for individuals with special needs has driven the development of LPWAN-based solutions that address issues like the limited range, high power consumption, and expensive hardware. To improve the reliability and reduce the latency, we propose an LPWAN-based emergency response system for people with special needs on a smart campus. The “smart campus” concept involves integrating smart technologies with physical structures to expand facilities, make decisions, and ensure campus sustainability. Smart classrooms, facial recognition-based attendance systems, and other small-scale solutions have been implemented on campuses. However, there is still no universal model for smart campuses, and the educational industry continues to benefit from digital technologies and smart applications that control various appliances and networked devices [5,12,13,14]. The proposed emergency response system can be utilized by anyone, including regular individuals. Our focus on people with special needs, particularly the visually impaired, stems from the recognition that they may encounter more challenges and face higher risks in emergency situations within a smart campus environment.

This research work makes three main contributions:

- We establish a framework for implementing an LPWAN-based emergency response system for people with special needs on smart campuses.
- We deploy Heltec WiFi LoRa 32 devices to improve communication and enable individuals with special needs to exchange information with their family and caregivers regarding their safety in the case of a risky or dangerous situation.
- We evaluate the performance of the proposed framework in terms of the latency and reliability.

The rest of the paper is structured as follows: Section 2 covers the related work. Section 3 highlights the background knowledge of LPWAN technologies. Section 4 explains the system model and design. Section 5 describes the experiments and results. Section 6 presents a discussion on the results, and finally, Section 7 concludes the paper.

## 2. Related Work

In this section, we provide an overview of existing research related to the LPWAN-based emergency response system proposed in this study.

The incorporation of technology into emergency response systems is important, and the IoT holds significant potential for supplying sensitive information during rescue and relief efforts in the event of natural disasters. While the LPWAN has been successfully integrated into various intelligent environments like smart buildings, banks, agriculture, smart vehicles, traffic management, smart cities, healthcare, and sensitive infrastructure [5,8,9,12,13,14], its implementation has not been geared toward providing LPWAN-based emergency response systems for individuals with special needs on a smart campus. Furthermore, ad hoc networks have not been utilized to reduce the latency and enhance the reliability. To address these concerns, we propose an LPWAN-based emergency response system that caters to people with special needs on the smart campus, using Hazara University in Pakistan as a case study.

According to research conducted by [15], there are specific requirements for an efficient emergency rescue and response mechanism for oceanic disasters, along with obstacles encountered when attempting to implement disaster management solutions in such situations. The authors suggest an approach based on the IoT that can be integrated with the existing oceanic network architecture to provide contextual information about the fishermen and their vessels. This approach provides partial context-awareness and can help address the challenges. This information can help with search and rescue operations by detecting any unusual activity on the fishing vessels. The study emphasizes the significance of technology acceptance and community participation in successfully adopting such an approach. By leveraging the advantages of the large-scale deployment of IoT solutions, coupled with community involvement, it is possible to bring about positive transformations in the community, which can be extremely beneficial in managing disasters in the ocean.

The authors of [16] suggested a model that employs Dragino shields to implement LoRa wireless communication technology for localization. This model utilizes Arduino microcontrollers to incorporate additional sensing mechanisms, improving the localization accuracy and reducing the power consumption in GPS-less environments. The experiments conducted in outdoor environments yielded positive outcomes, and the low power consumption and easy integration of localized environmental sensory data make LoRa technology a valuable resource for emergency services. In [17], the authors presented a WiFi-enabled microcontroller system that interfaces with several sensors, with the number of sensors scalable but limited by the microcontroller’s capabilities. The collected data are stored and visualized using the cloud platform Thinger.io, with endpoints available for sending emails and pushing data to external services. The system also uses the web-based service IFTTT to send alerts with a full emergency response profile to responders during emergency situations. This smart emergency response system is designed for use by individuals such as athletes, people with disabilities, and the elderly. The authors of [18] presented an assistive system called the “Online Blind Assistive System using Object Recognition”, which enables individuals with visual impairments to traverse unknown indoor and outdoor areas using an object recognition system. The implementation of a DNN using Python proved to be an accurate tool for object identification. However, the speed of the network may be a potential issue.

In [19], the authors introduced a mobile emergency response system to reduce the response time in the gas and oil industries. Ensuring low latency and robust security measures has been a critical challenge in emergency response, as any delay in the response time may have severe consequences and can also potentially result in data breaches in the case of cyber-attacks or theft. Informing people about emergencies needs to be faster. The authors proposed a method for controlling the motion in SNOW (Sensor Network Over White Spaces) in [20], which is an LPWAN operating in TV white spaces. This LPWAN utilizes Orthogonal Frequency-Division Multiplexing (OFDM) for synchronous communication between a base station and numerous low-power nodes. However, inter-carrier interference (ICI) can be an issue due to the OFDM-based design. Nevertheless, the OFDM-based architecture of SNOW leads to more pronounced inter-carrier interference (ICI). Hence, the authors put forward a solution for handling motion in SNOW to mitigate the impact of ICI and enhance the network’s overall functionality. In [21], the authors conducted an investigational analysis to examine the impact of mobility on LPWAN performance in mobile IoT applications. The study revealed that even minor mobility, such as human movement, has a significant impact on LPWANs. The authors found that mobility in various scenarios, such as the gateway’s location, the speed of the vehicle, and the placement of the end devices, can have a considerable effect on the LPWAN performance.

In [22], the authors investigated the mobility management of LPWAN systems and proposed a new IPv6-based solution to ensure communication continuity during changes in connection layer technology. This solution enables users to migrate between different LPWA networks and technologies, while optimizing the communication paths and preserving the bandwidth to reduce the data transfer time. The proposed method was validated through simulations and testing of the end-device handovers between LoRaWAN and NB-IoT. To assist first responders, a real-time communication system has been developed [23]. The centralized data hub known as the First Responder Units (FRUs) collects and distributes data from numerous connected peripherals. Additionally, a mobile emergency operations center (MEOC) is equipped and deployed with essential communication tools such as visual display devices and computers to provide support to first responders during their actions. The author of [24] presented the Emergency Communication System (ECS), which uses LoRa technology to provide infrastructure-free phone-based networks with long-range D2D communication. The LOCATE system comprises a mobile application that allows users to input critical emergency-related information and a distribution protocol that delivers emergency requests via multi-hop LoRa networks.

We summarized the related work in Table 1, and based on existing studies, we proposed research that focuses on the integration of IoT technology, particularly LPWANs, into emergency response systems. A specific emphasis is placed on addressing the needs of individuals with special requirements within smart campus environments. While the existing literature highlights the potential of the IoT, including LPWANs, in various sectors, such as disaster management, healthcare, and mobility, few studies have delved into the application of LPWANs for emergency response systems tailored to smart campuses. Prior work has explored diverse aspects of IoT technologies, including LoRa-based communication for localization, assistive systems for the visually impaired, and real-time communication systems for first responders.

Smart campus emergency response systems for individuals with special needs are worth considering to promote quality education for individuals with special needs by ensuring their safety and well-being. Several solutions have been proposed to address such problems, but many of these solutions come with significant limitations:

- IoT-Based Emergency Response Systems: Existing studies have discussed the potential of IoT technologies in emergency response systems. These networks have a significant role in the response and coordination of relief efforts for emergencies by using sensors, actuators, and communication networks. Nevertheless, IoT-based systems will have pros and cons, such as real-time monitoring and alerts, but they will also experience problems of scalability, reliability, and interoperability. In addition, IoT deployment and maintenance can be a burden and a challenge to the implementation of the technology, which may, in turn, hamper its acceptance, especially when resources are scarce.
- LPWAN-Based Solutions: The characteristic of LPWAN (Low-Power Wide-Area Network) technologies including LoRaWAN and NB-IoT is their long-distance data transmission with low power consumption, which makes them attractive to use. LPWAN-based emergency response systems can be considered a better solution as they are characterized by wide-range coverage and low energy consumption, which makes them suitable for a smart campus environment. However, they may be constrained by several problems, like network congestion, latency, and data throughput, particularly in areas with a high density of inhabitants and when there is a high demand.
- Assistive Technologies for Special Needs Individuals: Several efforts have been undertaken to design user-friendly and individualized assistive devices that can serve people who have disabilities, such as those with visual impairments. This technology, in the form of wearable devices, mobile applications, or sensory aids, is aimed at improving accessibility and safety. On the other hand, assistive technologies may be disadvantageous in terms of the usability, affordability, and compatibility with the existing infrastructure.
- Real-Time Communication Systems: During times of emergencies, real-time communication systems will allow the quick sending of critical data for the first responders and the people who are affected. The existing networks are often wirelessly linked via mobile apps and centralized data hubs in order to provide communication and coordination. Instant communication systems do provide quick response features, but they may also have some challenges, such as network congestion, privacy risks, and dependence on the internet, which can be interrupted during emergencies.
- Localization and Tracking Solutions: Localization and tracking technologies such as GPS and RFID can locate the position of individuals in the event of an emergency. Thus, the optimal use of resources and the best way of organizing the evacuation can be ensured. However, GPS-less environments or poor GPS reception may pose limitations to the indoor localization technologies, which might negatively affect their accuracy and reliability.

The existing studies primarily focus on specific applications or technical aspects, whereas our research aims to bridge the gap by proposing and evaluating an LPWAN-based emergency response system specifically designed for smart campuses, with a particular focus on addressing the needs of visually impaired individuals.

## 3. LPWAN Technologies

An LPWAN is a wireless communication technology that enables low-power devices with limited bandwidth to transmit data over long distances using low data rates. It is designed to support M2M and IoT networks and is typically more cost-effective and power-efficient than traditional mobile networks. This technology can support a large number of devices over a wide geographical area. LPWANs are classified into two main groups based on their protocols and technologies. The initial set of LPWAN technologies operates on unlicensed frequency bands and does not require cellular infrastructure. This group includes protocols such as LoRaWAN and Sigfox. The second group uses cellular technologies based on 3GPP standards and uses the licensed spectrum of mobile operators.

There are three types of cellular-based LPWANs: LTE-M, NB-IoT, and EC-GSM-IoT. LTE-M supports applications that require low to medium bandwidth and can achieve data rates of up to 1 Mbps. NB-IoT is optimized for low power consumption, long battery life, and deep indoor penetration, with data rates of up to 250 Kbps. EC-GSM-IoT is a modification of the GSM standard that offers extended coverage and better indoor penetration for IoT devices, with data rates of up to 240 Kbps. The choice of LPWAN technology and protocol depends on various factors, such as the application requirements, coverage area, data rate, power consumption, and network deployment cost.

Non-cellular LPWANs such as LoRaWAN and Sigfox are well-suited for applications that require low power consumption, long-range coverage, and low-cost deployment [25,26]. Cellular-based LPWANs such as LTE-M, NB-IoT, and EC-GSM-IoT are ideal for applications that require higher data rates, deeper indoor penetration, and better network reliability, but they may come with higher deployment costs [27,28]. Therefore, it is important to evaluate the advantages and disadvantages of each LPWAN technology and protocol before selecting the most appropriate one for a given application. Non-cellular LPWANs, such as LoRaWAN and Sigfox, operate on unlicensed spectrum bands and support devices with low power consumption and low data rates. They have a wide coverage range of up to 10 km in urban areas and up to 30 km in rural areas. The cellular-based LPWANs, on the other hand, utilize the licensed spectrum of mobile operators and provide a more reliable and secure connection. LTE-M is designed to support higher data rates than non-cellular LPWANs and has better coverage in urban areas. NB-IoT is specifically designed for low-power, low-data-rate applications, while EC-GSM-IoT is designed to provide better coverage in rural areas. LPWAN technology offers a flexible and scalable solution for a wide range of IoT applications, providing low-cost, low-power, and long-range connectivity [15]. A comprehensive description of the LPWAN technologies is provided below:

***A.***  ***Sigfox***

Sigfox offers LPWAN solutions that operate in license-free sub-GHz bands across various regions, such as 433 MHz in Asia, 868 MHz in Europe, and 915 MHz in North America. The technology used in Sigfox involves base stations that are equipped with software-defined radios, which connect to back-end servers through an IP network. For uplink communication, terminal devices use a 100 Hz bandwidth (BPSK) modulation in a 100 Hz data band, taking advantage of the sub-GHz spectrum’s Ultra Narrow Band (UNB) to maximize the frequency band utilization with low noise levels. This leads to better receiver sensitivity and lower energy consumption. Although Sigfox originally only supported uplink communication, it later upgraded to two-way communication. However, regional regulations limit the number of notifications sent via the uplink to 140 messages, with a message size of 12 bytes. Only four messages per day are allowed on the downlink, and the base station cannot verify each uplink message. The payload size of the downlink message is eight bytes without authentication, and the reliability of the uplink communication improves with message resending and time variation. This simplified approach reduces the overall cost of the solution [14,27,29].

***B.***  ***NB-IOT***

The narrowband Internet of Things (NB-IoT) is an LPWAN technology that operates in licensed frequency bands and is compatible with LTE and GSM. It offers three operational modes: Standalone, Guard Band, and In-Band, with a bandwidth of 200 kHz. The Standalone mode is intended to work with the current GSM frequency spectrum, while the Guard Band mode utilizes the inactive resource block of the LTE carrier. In contrast, the In-Band mode uses the resource block in the LTE carrier [30,31]. The NB-IoT communication protocols offer a range of benefits for IoT devices and their applications, as they are based on LTE concepts. Despite the limitation on the integration of BPSK and QPSK functions, NB-IoT carriers can support up to 100,000 appliances, making them a highly scalable option [14]. Using frequency division multiple access (FDMA), up to 20 kbps of data can be transmitted from a node to a base station, while Orthogonal Frequency Division Multi-Access (OFDMA) enables a maximum throughput range of 200 kbps to 1600 bytes for downlink connections. As such, NB-IoT represents a reliable and efficient solution for connecting IoT devices to the network, offering improved performance and functionality [32,33].

***C.***  ***LoRaWAN***

LoRaWAN is a spread-spectrum technology that uses the chirp spread spectrum (CSS) to modify signals in the sub-GHz range for two-way communication. This LPWAN technology is based on the LoRa standard that was introduced by Semtech in 2015 and was later standardized by the LoRa Alliance [34]. The signal model is tailored to accommodate channel noise and interference, and the transmitter generates a chirp signal that causes the frequencies of symbols to fluctuate over time while keeping their phases constant. The receiver can decrypt the chirp signal by varying the frequency shift, resulting in improved chipping symbols for each chirp signal. Furthermore, forward error correction (FEC) is employed to enhance the receiver’s sensitivity. LoRa technology boasts an impressive range of 1–30 km across both land and sea [35,36,37]. LoRaWAN, an open standard network stack, capitalizes on the physical layer characteristics of LoRa. Its objective is to enable sensors to exchange data frames through a server at a low data rate with relatively independent time intervals between transfers, such as one transmission per hour or per day. The network structure follows a star-star topology (GW), and the endpoints are connected to the network servers (NS) via the gateway. LoRaWAN adjusts its bitrate according to the quality of the available channels. LoRaWAN utilizes the SF (spreading factor) function to adjust the signal and battery strength. If the sensor node encounters poor connection quality, LoRaWAN increases the SF to transmit the modeled signal over longer distances, but this results in a lower bitrate. The transition is controlled by the LoRaWAN parameter DR (data rate), which ranges from DR0 (low bitrate, SF12) to DR5 in the EU (high bitrate, SF7) [38,39,40,41].

***D.***  ***TELENSA***

Telensa, a UK-based firm, offers IoT-based private and public network solutions that cater to smart cities, smart metering, and smart lighting needs. The company has successfully installed millions of linked street lighting systems across different countries and employs IoT technology for tracking, detection, and monitoring in smart cities and homes. Telensa’s technology is similar to that of Sigfox and is widely used to regulate millions of lights and other products. Recently, Telensa has been focused on creating innovative applications for smart cities, such as urban data insight and urban IQ, using next-generation light pole sensors that employ artificial intelligence technology. These multi-sensor pods use the latest smartphone artificial intelligence capabilities and automobile camera and radar-imaging technology to provide a comprehensive understanding of road infrastructure. In addition, Telensa is leading an urban data initiative that aims to change the way data are collected and utilized by local governments, creating what they call a “digital twin of a city”. Telensa has designed a modulation mechanism specifically for the UNB (Ultra Narrow Band) that enables low-data-rate wireless connections between end nodes and base stations operating in the unlicensed sub-GHz ISM (Industrial, Scientific, and Medical) band. UNB technology is designed for low-bandwidth transmission, and Telensa has developed a vertical grid stack for its network, which allows for easy integration with third-party software and end-to-end solutions for LPWAN applications. The company has more than 8 million UNBs operating in 30 countries, and its technology is low risk and top-notch. To ensure seamless integration into applications, the company is standardizing its technology to meet ETSI (LTN) network requirements. Telensa’s UNB device has an uplink rate of 62.5 bps and a downstream rate of 500 bps. The company is working with Sigfox in the ETSI LTN group [27].

***E.***  ***INGENU RPMA***

INGENU, formerly known as On-Ramp Wireless, uses a patented LPWAN technology that differs from many other LPWAN technologies on the market by operating in the 2.4 GHz ISM band instead of the sub-GHz band. INGENU’s LPWAN technology has the advantage of improved throughput and capacity in regions where the work-cycle maximum regulations do not limit the 2.4 GHz band. Additionally, the Random Phase Multiple Access (RPMA) physical access technique used by INGENU’s LPWAN technology is a distinctive feature that differentiates it from other LPWAN technologies. RPMA employs a wide range of direct sequences for uplink communication, enabling multiple transmitters to use a single time slot. This form of code division multiple access (CDMA) increases the signal-to-interference ratio for each link by ensuring that the channel does not align the transmitters exactly at the same time. The exceptional receptor sensitivity of −142 dB and a bond budget of 168 dB make RPMA unmatched in the industry. Multiple demodulators on the receiving end of base stations decode signals that arrive at different times in a slot. While there may be some link asymmetry, INGENU’s technology enables two-way communication. Downlink communication is accomplished by base stations transmitting signals to specific end devices, which are subsequently sent utilizing CDMA. The end nodes can also modify their broadcast strength to reach the nearest base station without interfering with surrounding devices. INGENU is leading the way in standardizing the physical layer requirements according to the IEEE 802.15.4k, with RPMA technology developed in compliance with these requirements [27].

Table 2 presents a comparison of the primary technical features of various LPWAN technologies, such as the availability, bandwidth, link budget, battery life, energy usage, frequency, security, and private network allocation [27,34]. The appropriate LPWAN technology for IoT applications is determined by these key factors. Telensa, Sigfox, and NB-IoT do not support private network deployment as the signal strength relies on the service provider base stations, while RPMA and LoRa enable the creation of private networks for these applications. Telensa, Sigfox, and LoRaWAN utilize sub-GHz frequencies that are not licensed, and their communication capabilities are designed to overcome multi-path interference and fading. This allows them to provide effective communication. In contrast, NB-IoT uses licensed frequencies to provide high-quality service, but at a higher cost. However, NB-IoT devices have a shorter service life due to the OFDM/FDMA approach, which requires a higher current for simultaneous communication from its terminal tools. Despite being released in 2016, the NB-IoT standard is still in its supply phase, while other LPWAN deployment models have already matured. At present, LoRaWAN is the most widely used LPWAN technology, with deployments in over 160 countries. LoRaWAN can establish public networks, local area networks, and a hybrid operating model that combines a local LoRaWAN network with a base station public network. LoRa uses unlicensed frequencies, is energy-efficient and affordable, and offers a communication range of 1–30 km over ground and water, respectively [27,34].

## 4. System Model and Design

**Figure 1:**
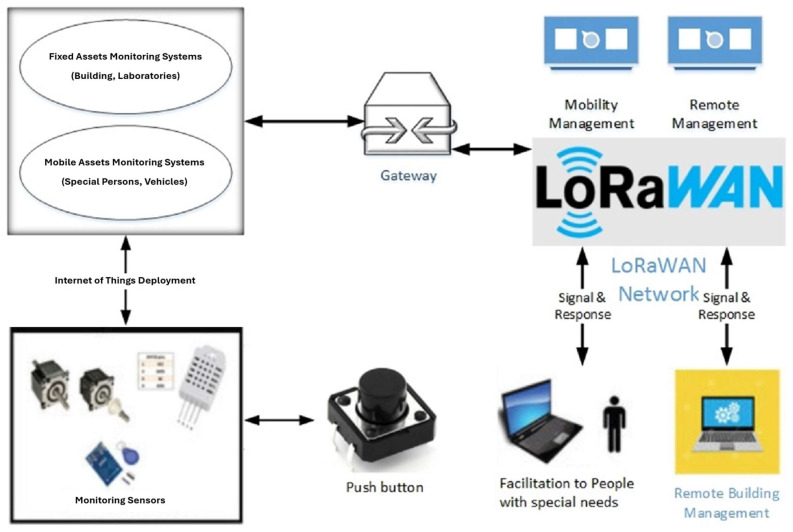
An LPWAN-based emergency response system for individuals with special needs in smart buildings.

In our study, we designed an LPWAN-based emergency response (LBER) system to meet the needs of people with special requirements on a smart campus. To ensure data distribution in areas with WiFi coverage and to fill gaps in areas without, we utilized WiFi as the primary means of communication and LoRaWAN as a backup. In the event of an emergency, WiFi may become unreliable and the electricity supply may be disrupted, but the LoRaWAN network will remain functional. Our solution utilizes two Heltec WiFi LoRa 32 V2 devices, each equipped with BLE, WiFi, and LoRa technology with one serving as the sender and the other as the receiver of data. To initiate an emergency response, the user simply presses a panic button, and the message is transmitted to the recipient. This approach allows for the reduced latency and improved reliability of the emergency response system on a smart campus. Figure 1 provides a visual representation of our proposed model to facilitate better understanding.

***A.***  ***WiFi LoRa***

**Figure 2:**
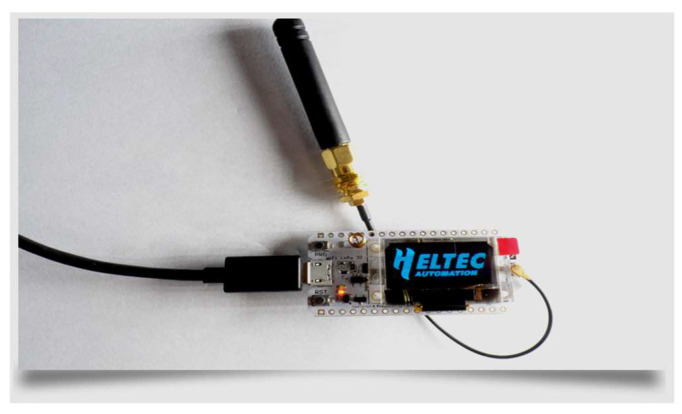
Heltec WiFi LoRa 32 board [42].

The WiFi LoRa 32 V2, a well-known IoT development board, is produced by Heltec Automation (TM) and is depicted in Figure 2. This highly integrated board is based on ESP32 + SX127x and has WiFi, Bluetooth Low Energy (BLE), and LoRa capabilities, as well as a Li-Po battery management system and a 0.96″ Organic Light-Emitting Diode (OLED). It is an extremely low-power solution board that is ideal for IoT creators working on projects such as smart cities, smart farms, and smart homes [25].

The ESP32 chip has the capability to support the Transmission Control Protocol/Internet Protocol (TCP/IP), the 802.11 b/g/n WiFi Medium Access Control (MAC) protocol, and the WiFi Direct specification. Meanwhile, the SX127647 is a half-duplex transceiver that operates at a low intermediate frequency and utilizes Semtech’s patented LoRa modulation technique, along with a low-cost crystal, to achieve a sensitivity level that surpasses −148 dBm. It is pertinent to mention that the sensitivity depends on the spreading factor and bandwidth. Additionally, the SX1276 has two modems—one for Frequency Shift Keying (FSK) and one for LoRa spread spectrum modulation—which can be utilized based on the selected modes. By utilizing the LoRa modulation technique, the device can gain greater immunity to in-band interference.

***B.***  ***GPS Module***

**Figure 3:**
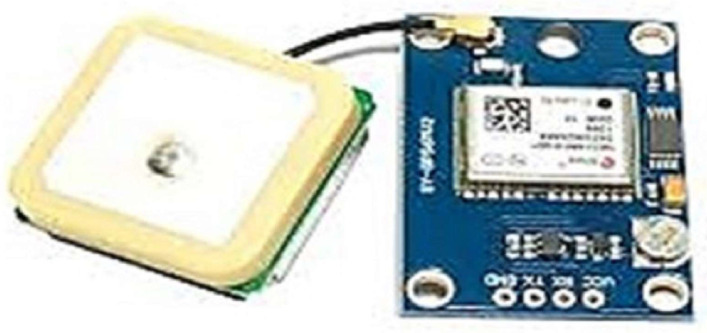
GPS NEO-6M u-blox module.

The GPS module is an adaptable and economical positioning device designed for wide-ranging applications. It offers a 2.5 m horizontal positioning accuracy and has the capability to store the device’s configuration when it is powered off. It is equipped with an IPX interface for attaching various active antennas that provide a strong signal, and it has 50 channels for signal reception. It features four-pin connections for VCC, GND, TX, and RX. Customized settings can be created using the u-center software provided by u-blox AG [26]. The module is primarily utilized for satellite navigation, and it can determine speed and location on land, air, and sea and provide precise maps, tracking systems, navigation, and aircraft positioning. NEO-6 modules are particularly suitable for mobile devices that have strict limitations on cost and space due to their compact design and power and memory options. The specifications of the GPS module are listed in Table 3 and Figure 3 illustrates the GPS module.

***C.***  ***Panic Button***

**Figure 4:**
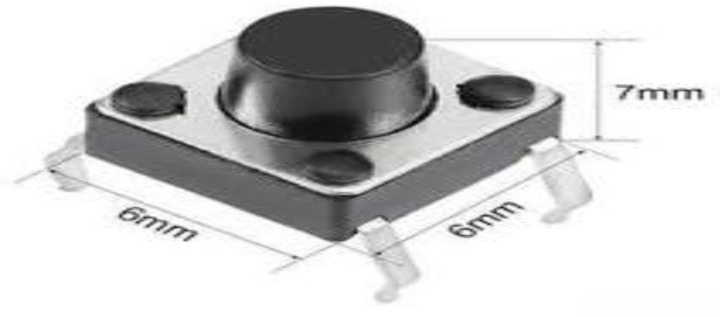
Push button.

The panic button proposed in this study is designed to have the utmost effectiveness and efficiency as it is intended to be used in emergency or critical situations by individuals with special needs. The Wi-Fi LoRa 32 v2-based IoT panic button will be set up to address the needs of individuals with special needs who may be faced with various risks, including panic situations, fear of theft, or difficulty navigating due to blindness. Many of these individuals may lack technical proficiency in operating devices, hence necessitating the need for a simple and reliable device that can alert someone when help is needed. This IoT panic button will be enabled over LoRa, and we are using the WiFi LoRa 32 module to increase its portability and simplicity. The operation of this panic or push button is straightforward, requiring the user to push the button to trigger an alert, which will immediately send their location to the designated person. Figure 4 depicts the design of the push or panic button.

There are several benefits of implementing a panic button, including but not limited to the following:

1. The ability to swiftly inform family members or relevant authorities in case of an emergency.
2. Expedite rescue efforts, thereby potentially saving a life.
3. Provide assistance to individuals facing potential risks.

## 5. Experiments, Results and Discussion

***A.***  ***Experimental Setup***

**Figure 5:**
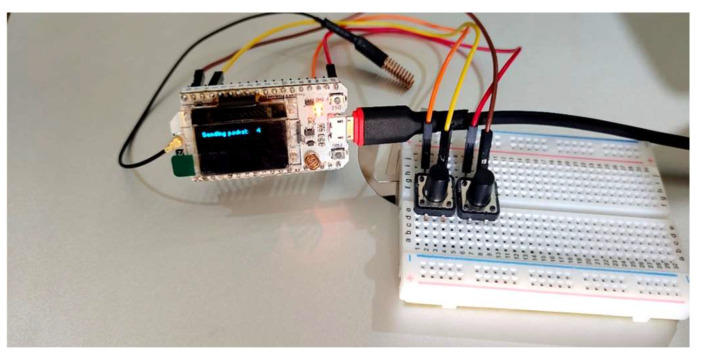
Experimental setup.

The experimental setup, as illustrated in Figure 5, involved ensuring the proper connection of all the components and devices according to the desired configuration. To enable the flow of current, the Heltec WiFi LoRa 32 development board utilized a Power Bank to receive external power. In the event of danger, the visually impaired person would activate the panic button. Subsequently, the GPS module would provide the location of the danger and send an alert to a designated emergency care provider.

In our experimentation, we utilized LoRa modulation with specific transmission parameters, including the spreading factor (SF), bandwidth (BW), and coding rate (CR). For the spreading factor, we employed a default value throughout the trials, which was 7. The bandwidth was set at 125 kHz, and the coding rate was fixed at 4/5. As for the number of packets sent for each testing setup, we transmitted a total of 50 packets at each distance in every experiment, maintaining consistency across all the trials.

***B.***  ***Results and Discussion***

**Figure 6:**
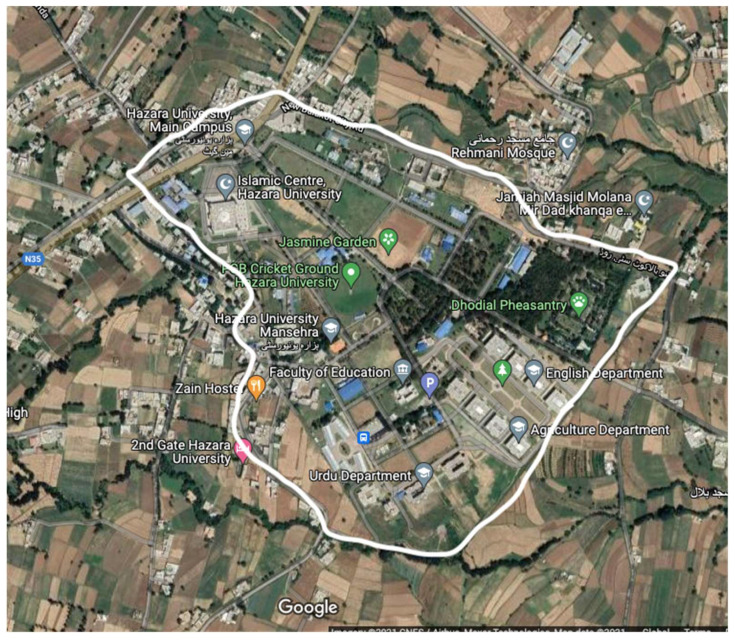
The morphology of the testing site shown through a map.

To evaluate the efficacy of the system, a total of 18 experiments were executed, comprising 9 trials in an open field and an additional 9 in IT Building No. 3 at Hazara University in Mansehra, Pakistan. The morphology of the testing site, as depicted on a map, is illustrated in Figure 6. The selected open field was elevated and proved demanding to test the system’s performance in challenging environments. The experiments were conducted using three distinct antenna heights (0 m, 1 m, and 2 m) and three varied packet sizes (16 bytes, 32 bytes, and 64 bytes), while keeping the default TX power (dBm) of 15 for all the tests. The end device was tested at varying distances from 0 m to 600 m, with increments of 50 m. For each experiment, the antenna height was set at 0 m for the first experiment, 1 m for the second, and 2 m for the third experiment for a data packet size of 16 bytes. The same process was repeated for 32 and 64 bytes of data packets. To determine the system’s performance based on the packet size and antenna height, 50 packets were transmitted at each distance in each experiment, and the average of the experiment results was used to evaluate the performance.

***a.***  ***Reliability***

Reliability pertains to the capability of consistently performing with dependability and trustworthiness. In the context of a network system, reliability can be determined by evaluating the probability of successful interaction between every pair of nodes or by measuring the operational efficacy and signal strength (RSSI) following the experiment. The RSSI represents the received signal power in milliwatts, expressed in dBm, and is utilized to assess data transmission across different settings and distances. The packet delivery ratio (PDR) is calculated as the ratio of valid received packets to the total number of transmitted packets, as demonstrated in Equation (1). The PDR is an indicative measure of communication reliability, which can be used in tandem with the RSSI to provide a comprehensive assessment of system performance. The RSSI value can be utilized to gauge the strength of the signal received by the receiver from the sender, and it is represented as a negative value, where a value closer to 0 signifies a stronger signal.

(1) $$\mathrm{PDR}=\frac{R}{S}$$

*R*: Total packets received successfully
*S*: Total packets sent.

**Figure 7:**
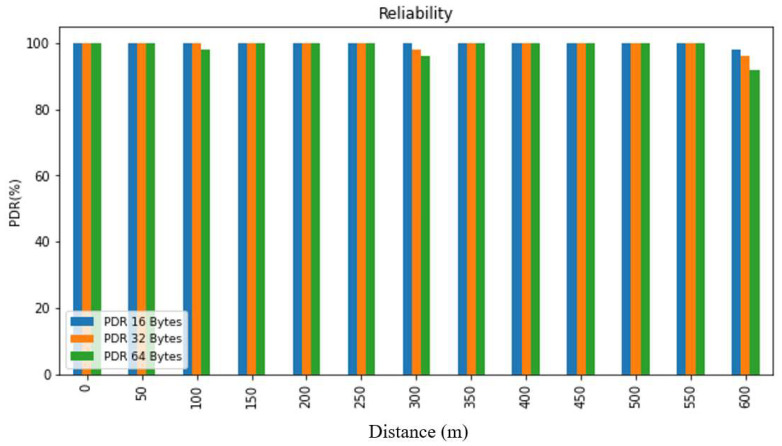
PDR according to distance and packet size when antenna height = 0 m.

**Figure 8:**
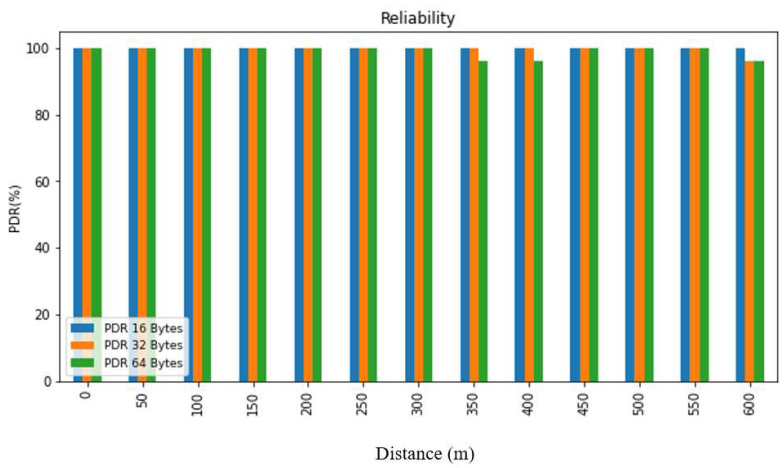
PDR according to distance and packet size when antenna height = 1 m.

**Figure 9:**
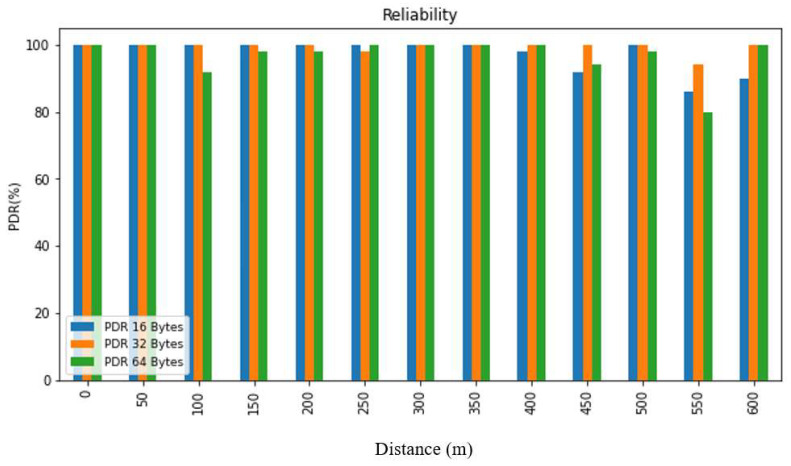
PDR according to distance and packet size when antenna height = 2 m.

**Figure 10:**
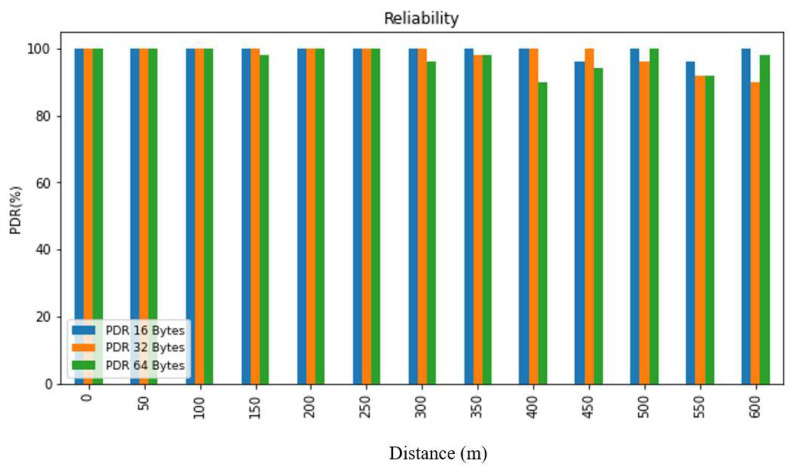
Reliability analysis of the first floor.

**Figure 11:**
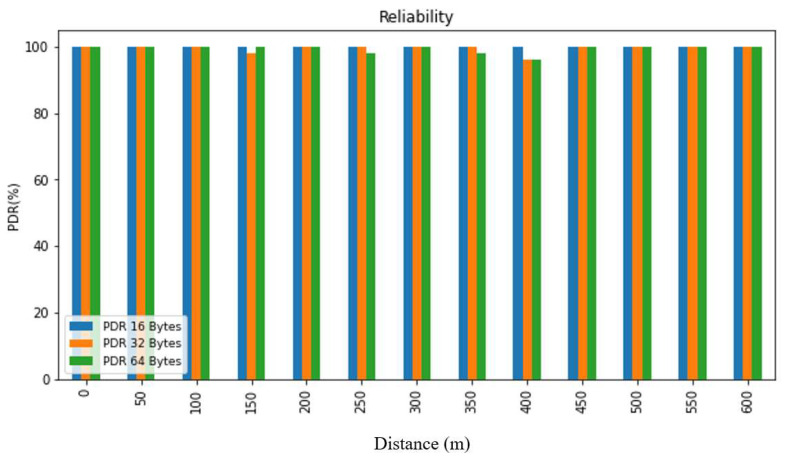
Reliability analysis of the second floor.

**Figure 12:**
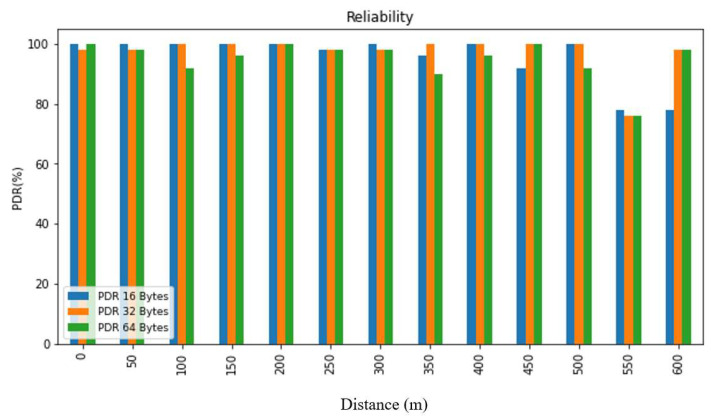
Reliability analysis of the third floor.

In order to evaluate the collected data, we first summarized it and then presented it using figures and tables. Figure 7, Figure 8, Figure 9, Figure 10, Figure 11 and Figure 12, along with Table 4, Table 5, Table 6 and Table 7, display the reliability and RSSI for various antenna heights and distances. These visuals allow us to observe both high and low reliability and RSSI values. The experiment was conducted at Hazara University, covering an area of 1300 m^2^, and yielded excellent results. We focused on the reliability data for a distance of 600 m in two different locations.

i. The transmitter was positioned on the left side of a location, specifically Boys Hostel No. 2, situated in an unobstructed area.
ii. The transmitter was initially deployed outside IT Building No. 3 to extend the range of transmission to the receiver.

Hazara University is a verdant campus with an abundance of trees that form dense forests, and the central IT Building is surrounded by towering trees, which pose a significant impediment to D2D communication. To evaluate LoRa’s reliability performance over varying distances, the receiver was placed at different distances ranging from 0 to 600 m in increments of 50 m, denoted as R0 to R600, with the transmitter fixed at one end. For each distance, 50 trials were conducted to determine the reliability, with 3 different packet sizes of 16, 32, and 64 bytes, and 3 different antenna heights of 0, 1, and 2 m in an open area and on the first, second, and third floors of IT Building 3.

This study’s findings showed that the reliability of the system was affected by the distance, payload size, and antenna height. The reliability was found to decrease with an increase in the distance and payload size due to the longer airtime in LoRa transmission, while it increased with an increase in the antenna height. This study evaluated the reliability for different antenna heights and packet sizes and found that it was lowest for low antenna heights and large distances and highest for high antenna heights and small distances. Additionally, the reliability increased as the distance and packet size decreased. This study also found that the LoRa transmission range decreased with a decreasing antenna height in the case of indoor D2D-LoRa networks. Despite some distances exhibiting lower reliability due to high obstacles, the reliability results were stronger than expected, with more than 98% reliability demonstrated at all the distances and antenna heights in all the settings. As evident from the presented tables and figures, all the environments exhibited more than 98% reliability at all the distances and antenna heights, except for a few distances with high obstacles. Compared to similar studies [1,12,17], the measured distances and reliability results were remarkably strong.

***b.***  ***Latency***

Latency, the amount of time that has passed since an event, is an important factor in network performance, particularly in terms of the time it takes for data to travel from one point to another. This duration, commonly referred to as “round trip delay”, encompasses both the transmission and reception times. The latency is calculated by summing together all sorts of delays, including transmitting and receiving delays. It is measured in ms [43]. Equation (2) is used to compute the latency.

(2) $$Latency={REC}_{T}-S_{T}$$

**Figure 13:**
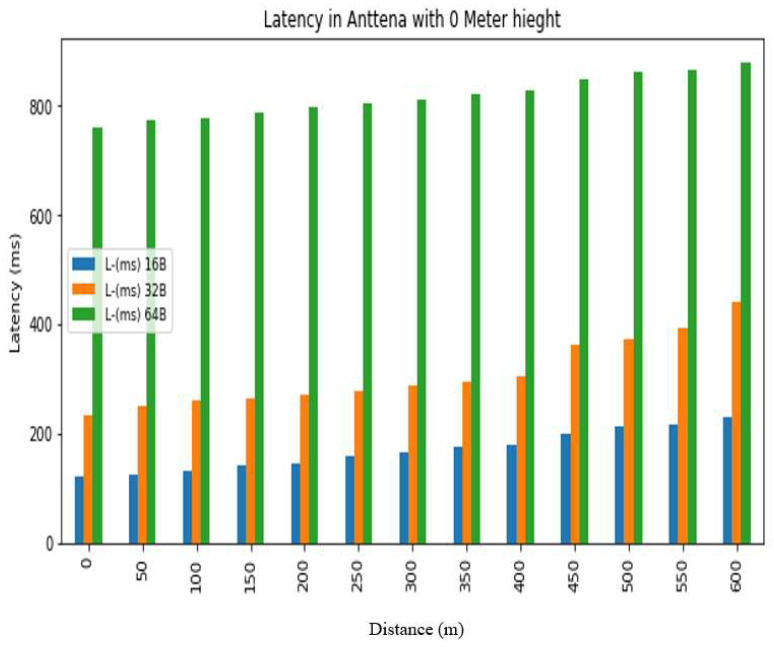
Latency analysis for antenna height = 0 m.

**Figure 14:**
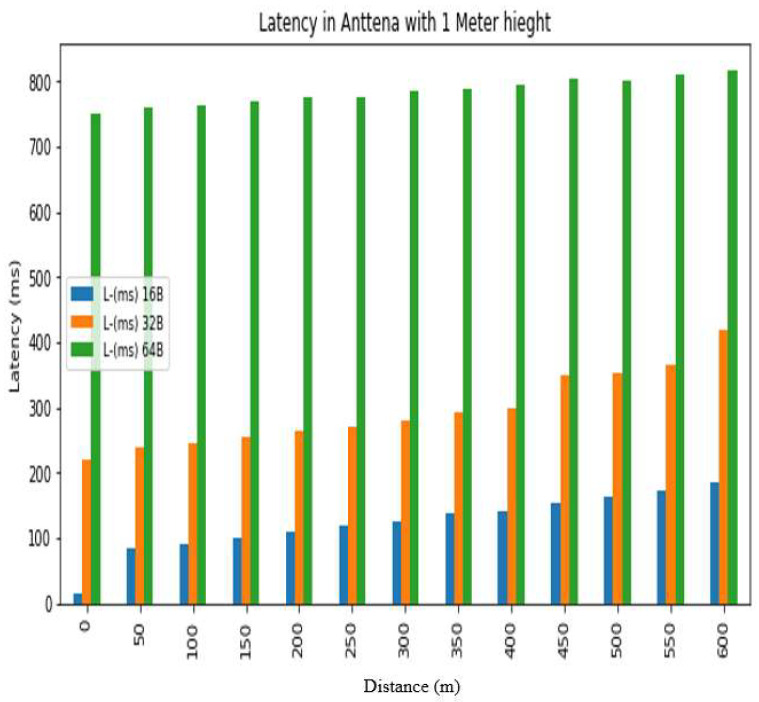
Latency analysis for antenna height = 1 m.

**Figure 15:**
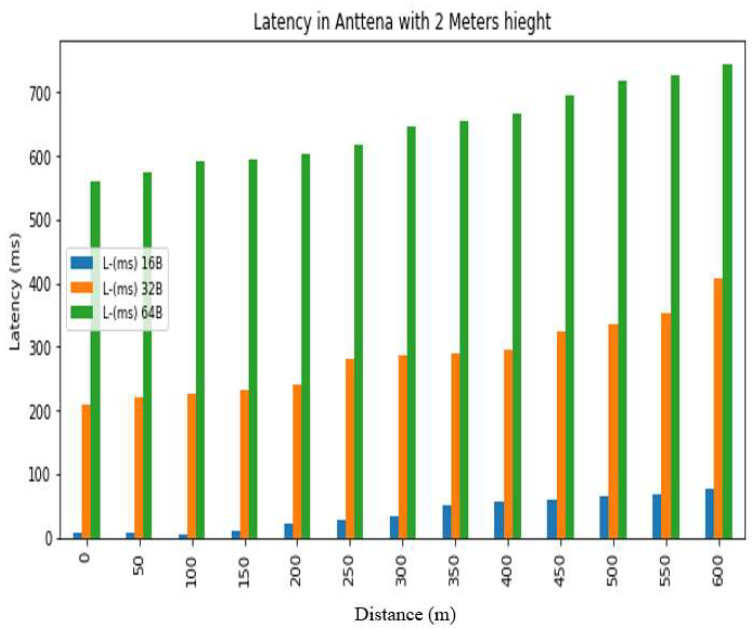
Latency analysis for antenna height = 2 m.

**Figure 16:**
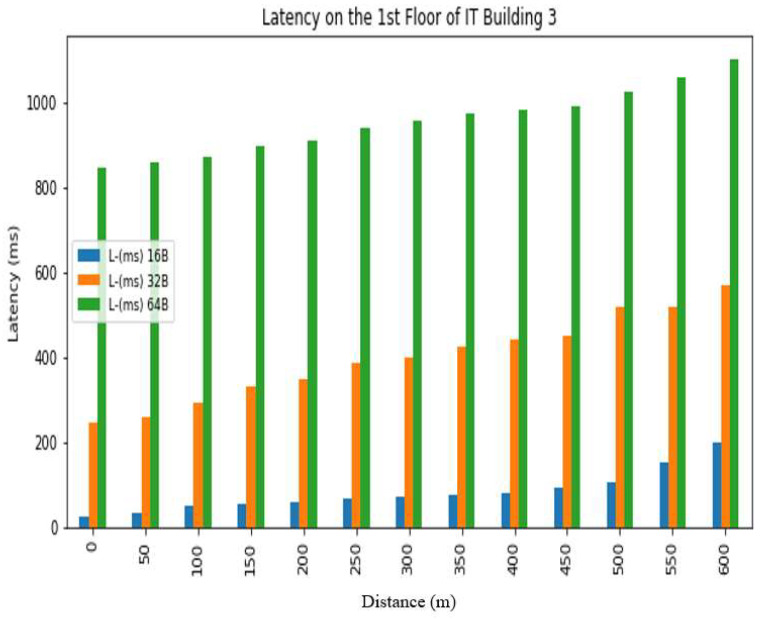
Latency analysis on 1st floor.

**Figure 17:**
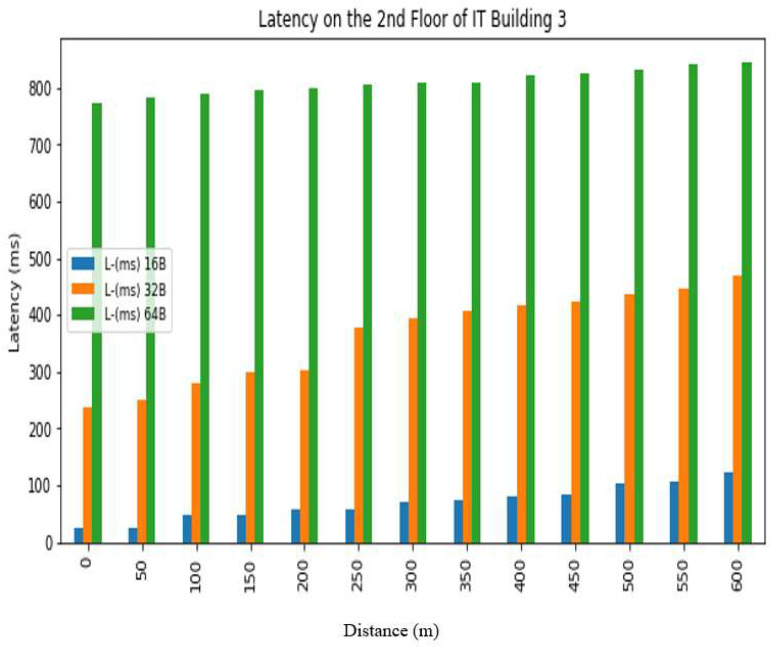
Latency analysis on 2nd floor.

**Figure 18:**
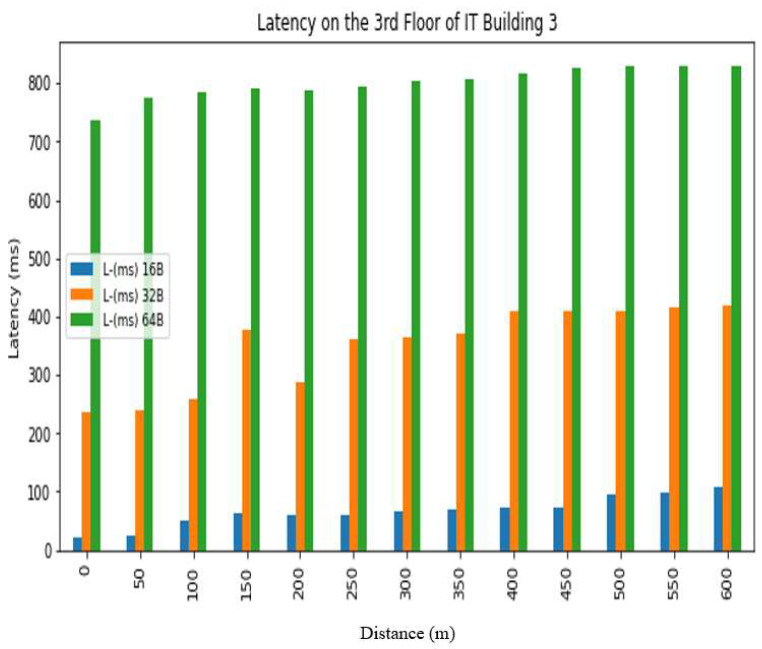
Latency analysis on 3rd floor.

The *REC_T_* denotes the receiving time and the sending time is denoted by *S_T_*. The latency was evaluated in a test conducted at Hazara University, in which the receiver was positioned at various distances from the transmitter, located on the left side of Boys Hostel 2, to assess LoRa’s performance at different ranges. The results are shown in Figure 13, Figure 14, Figure 15, Figure 16, Figure 17 and Figure 18, along with Table 8 and Table 9. The distances between the receiver and transmitter ranged from 0 m to 600 m, with a 50 m interval between them, represented as R0, R50, R100, R150 ... R600. The transmitter remained stationary, while the receiver was gradually moved away from it at the distances mentioned above. The results demonstrate that the latency of the LoRa transmission increases significantly as the payload size increases. For example, when the payload size increases from 16 bytes to 32 bytes and then to 64 bytes, the average latency increases by 8% and 17%, respectively, at 600 m and an antenna height of 0 m. Similarly, at 600 m and an antenna height of 0 m, the latency for 64 bytes of data is more than twice that of 16 bytes of data. This indicates that larger payload sizes may not be suitable for applications that require low latency, and smaller packet sizes should be used for low-latency applications. The experimental results demonstrate that the performance of LoRa in terms of the reliability and latency is affected by the distance, payload size, and antenna height. The results show that increasing the antenna height leads to higher reliability and lower latency, while larger payload sizes and longer distances result in lower reliability and higher latency. These findings can be used to optimize the design of LoRa-based IoT systems for specific use cases and environments.

Numerous methods have been proposed in the literature to address wireless communication challenges during emergency response situations. However, these methods are limited in terms of the latency and reliability, particularly in densely populated areas like urban zones that are prone to natural disasters such as earthquakes, floods, and storms. For instance, communication systems based on WiFi and other radio technologies have shorter ranges, making it difficult to cover large areas such as those affected by urban emergencies. To address this research gap, we proposed an LPWAN-based emergency response system for people with special needs on a smart campus. To test the efficacy of the system, we conducted real-world experiments at Hazara University using Heltec WiFi LoRa 32 devices. Our experiments involved varying the distance and antenna height above the ground surface. We found that LoRaWAN technology is a promising choice for improving communication on smart campuses. Our results revealed that a higher antenna size and minimum distance improved the reliability and decreased the latency, as signals could easily pass through small objects and obstacles. Conversely, a lower antenna size and maximum distance resulted in lower reliability and increased latency, owing to the urban and green campus environment with different types of obstacles.

## 6. Discussion

This study aimed to propose and test an LPWAN-based emergency response system for people with special needs on a smart campus and to compare the performance of the LoRa-based system with other wireless communication technologies in terms of the latency, reliability, and range. The analysis of the performance of the proposed research work has been based on the benchmark proposed in studies [1,12,17]. We evaluated the proposed work using two parameters: reliability and latency. For data transmission, we utilized the WiFi LoRa 32 board with payload sizes of 16, 32, and 64 bits, and the results were compared at different heights. Additionally, experiments were conducted in both open environments and environments with obstacles such as buildings and trees. The results of our experiments suggest that LoRaWAN technology is a promising choice for better communication in smart campus emergency response systems. Our experiments involved the deployment of Heltec WiFi LoRa 32 devices at Hazara University, where we conducted several experiments at varying distances and heights above the ground’s surface. We sent out 50 packets from each distance, with 50-m intervals in length, ranging from 0 to 600 m, and assessed their reception, RSSI, network latency, and reliability.

The experimental results indicate that the reliability and latency of LoRa-based communication systems are improved with a higher antenna size and minimum distance. This is because the signals are less likely to be obstructed by small objects and obstacles, resulting in better transmission quality. Conversely, with a lower antenna size and maximum distance, the reliability and latency suffer due to the green and urban environment of the campus, where signals face different types of objects and obstacles. It is worth noting that our results suggest that the performance of the LoRa-based system is not affected by cold and rainy weather, as the RSSI values approached zero, indicating a strong RSSI. This is in contrast to other cellular and satellite technologies, which are negatively affected by rainy weather. Thus, LoRaWAN technology appears to be more resilient in adverse weather conditions, making it a more reliable choice for emergency response systems.

We conducted extensive experiments to investigate the real-world implementations of LoRaWAN technology in the context of smart campus emergency response systems. By evaluating the performance of the proposed solution using these parameters, we aimed to provide insights into its practical applicability and effectiveness in addressing the unique challenges faced by individuals with special needs in emergency situations. Our study builds upon the existing literature by focusing specifically on LPWAN-based emergency response systems tailored for smart campuses, with a particular emphasis on addressing the needs of visually impaired individuals. We conducted experiments at Hazara University to assess the reliability and latency of LPWAN technology in various scenarios, including different distances and antenna heights. The findings from our experiments revealed that LPWAN technology, particularly LoRaWAN, presents a viable option for implementing emergency response systems in smart campus environments, with a significant reliability. Our study contributes to the existing body of knowledge by providing insights into the performance of LoRa-based communication systems under different environmental conditions, such as urban and green campus environments with various obstacles. We observed that factors like the distance, payload size, and antenna height significantly impact the reliability and latency of LoRa communication, which can inform the design and optimization of emergency response systems for specific use cases and environments.

Our study has some limitations that should be addressed in future research. Firstly, the experiment was conducted in a single location, and the findings may not be generalizable to other environments. Secondly, the study only tested the performance of the LoRa-based system in terms of the reliability and latency, and other factors, such as the energy consumption, should be considered in future studies. Finally, the experiment was conducted in a controlled environment, while real-world scenarios may have more complex network conditions that may affect the system’s performance. The experiments show that LoRaWAN technology is a promising choice for emergency response systems on smart campuses. The system’s reliability and latency are improved with a higher antenna size and minimum distance, making it a more resilient choice for emergency response in adverse weather conditions. Future research should focus on addressing the limitations of this study and exploring the system’s energy consumption and performance in real-world scenarios.

## 7. Conclusions

In this study, we successfully implemented an LPWAN-based emergency response system for people with special needs on the Hazara University campus, using Heltec WiFi LoRa 32 devices as the sender and receiver nodes. Our experimental evaluation of the system’s performance has revealed valuable insights into the impact of various factors, such as the distance, antenna height, and packet size, on the reliability and latency of LoRa communication in an indoor D2D network. One of the key challenges of the system implementation was the presence of large trees surrounding the academic block buildings, which presented obstacles for D2D communication. Despite this challenge, our results have shown that LoRa technology can be a promising choice for reliable communication in emergency response scenarios, even in the presence of environmental barriers. Moreover, we observed that the data transmission quality of LoRa was positively affected by cold and rainy weather, suggesting that it may be more resilient to adverse weather conditions than other cellular and satellite technologies. Our findings have also highlighted the critical role of the antenna height in determining the reliability and latency of LoRa communication. Specifically, we found that the reliability of the system was highest for high antenna heights and small distances, while the latency was lowest for high antenna heights and small distances. These results suggest that optimizing the antenna height can significantly improve the performance of LoRa-based emergency response systems in indoor settings.

This study has provided valuable insights into the design and optimization of LPWAN-based emergency response systems for people with special needs. Future research in this area could focus on investigating the scalability and generalizability of our findings to different types of indoor environments and emergency scenarios. Further exploration of alternative LPWAN technologies and hybrid communication approaches may help to address some of the limitations of LoRa in high-density node environments.

## Figures and Tables

**Table 1:** An overview of related works.

Reference	Contributions	Limitations
[15]	The authors implemented a partial context-aware algorithm to manage emergencies in the ocean by detecting any atypical movements of fishing vessels.	There is a problem of latency in determining the state of the vessel.
[16]	The proposed system was developed using Dragino LoRa v1.3 Shield devices, which are specifically designed to be easily programmed and integrated with an Arduino Uno microcontroller.	The system is not reliable because the system accuracy is not good.
[17]	The author connected several sensors to a microcontroller that is equipped with WiFi capabilities to detect various conditions, such as motion, vibration, and pulse rate. Additionally, a GPS module was integrated with the WiFi-enabled microcontroller to enable positioning and tracking functionalities.	The reliability of the system may be limited in certain environments where GPS signals cannot penetrate, such as underground or underwater, as GPS is used as part of the system.
[20]	The D-OFDM technique, which utilizes TV white spaces, was implemented by the authors. SNOW, a communication technology that permits extensive and simultaneous communication between multiple nodes and a base station (BS), was also utilized.	The hardware is very expensive.
[23]	A Mobile Emergency Operations Center (MEOC) was established to aid the activities of first responders and to provide support with the development of a real-time communication technology.	Neglecting the aspects of latency and reliability.

**Table 2:** Comparison of key technical specifications for LPWAN technologies [27,34].

Technologies	Sigfox	NB-IoT,	Telensa	LoRa	INGENU RPMA,
Creator	**Sigfox**	3 GPP	Telensa	Semtech	Ingenu
Modulation	BPSK	QPSK	UNB 2-FSK	CSS	UL: RPMA-DSSS
					DL: CDMA
Frequency	Sub-GHz ISM	Licensed LTE Frequency bands	Sub-GHz, ISM EU: 868 MHz US: 915 MHz AS: 430 MHz	Unlicensed ISM	2.4 GHz ISM
	EU 868 MHz			EU 868 MHz	
	US 902 MHz			US 915 MHz	
Data Rate.	100 bps (UL)	200 kbps	UL: 62.5 bps	0.3–50 kbps	UL: 624 kbps DL: 156 kbps
	600 bps (DL)		DL: 500 bps		
Energy consumption	Very low	Low	Low	Very low	High
Bandwidth	100 or 600 Hz DL: 1.5 kHz	180 kHz	100 kHz	125, 250, 500 kHz	1 MHz
Link budget (dB)	EU: 162	189	EU: 161	EU: 151	EU: 168
	US: 146		US: 149	US: 171	US: 180
Range	10 km (Urban)	1 km (Urban)	Urban: 3 km	1–30 km	Urban: 15 km
	50 km (Rural)	10 km (Rural)	Rural: 16 km		Rural: 48 km
Allow private networks	No	NO	NO	YES	YES

**Table 3:** GPS module specifications.

Parameters	Specification
**Operating Voltage**	2.7–5 V
**Baud Rate**	9600 (default)
**Update Rate for Navigation**	1 Hz (default) 5 Hz
**Tracking Sensitivity**	−161 dBm
**Operating Current**	45 Ma
**Communication Protocol**	NMEA (default), UBX library

**Table 4:** Reliability results for different distances and packet sizes when antenna height = 0 m.

Antenna Height = 0 m						
Distance	P-Size: 16 Bytes		P-Size: 32 Bytes		P-Size: 64 Bytes	
	PDR %	RSSI	PDR %	RSSI	PDR %	RSSI
0 m	100%	−22.0	100%	−27.42	100%	−35.18
50 m	100%	−81.0	100%	−92.82	100%	−99.80
100 m	100%	−92.3	100%	−99.24	98%	−104.54
150 m	100%	−95.6	100%	−94.84	100%	−92.48
200 m	100%	−106.9	100%	−99.98	100%	−102.00
250 m	100%	−103.3	100%	−99.98	100%	−106.34
300 m	100%	−108.9	98%	−111.62	96%	−121.92
350 m	100%	−114.9	100%	−111.64	100%	−120.20
400 m	100%	−118.2	100%	−114.00	100%	−127.42
450 m	100%	−115.9	100%	−115.60	100%	−131.00
500 m	100%	−122.6	100%	−111.66	100%	−120.70
550 m	100%	−120.6	100%	−113.00	100%	−121.50
600 m	98%	−130.9	96%	−123.00	92%	−135.60

**Table 5:** Reliability results for different distances and packet sizes when antenna height = 1 m.

Antenna Height = 1 m						
Distance	P-Size:16 Bytes		P-Size: 32 Bytes		P-Size: 64 Bytes	
	PDR %	RSSI	PDR %	RSSI	PDR %	RSSI
0 m	100%	−41.3	100%	−24.88	100%	−56.6
50 m	100%	−99.06	100%	−78.82	100%	−76.86
100 m	100%	−108	100%	−89.88	100%	−91
150 m	100%	−109.8	100%	−86.32	100%	−93.52
200 m	100%	−110.42	100%	−92.52	100%	−86.44
250 m	100%	−112.84	100%	−97.5	100%	−104.32
300 m	100%	−117.28	100%	−100.66	100%	−107.9
350 m	100%	−124.26	100%	−108.28	96%	−115.72
400 m	100%	−127	100%	−106.68	96%	−118.14
450 m	100%	−124.1	100%	−111.86	100%	−118.8
500 m	100%	−128.76	100%	−111.18	100%	−127.8
550 m	100%	−129.12	100%	−120	100%	−121.84
600 m	100%	−130.66	96%	−128.52	96%	−128.78

**Table 6:** Reliability results for different distances and packet sizes when antenna height = 2 m.

Antenna Height = 2 m						
Distance	P-Size:16 Bytes		P-Size: 32 Bytes		P-Size: 64 Bytes	
	PDR %	RSSI	PDR %	RSSI	PDR %	RSSI
0 m	100%	−48.16	100%	−42	100%	−55
50 m	100%	−85.86	100%	−84.00	100%	−77.10
100 m	100%	−86.58	100%	−96.48	100%	−86.72
150 m	100%	−89.8	98%	−92.8	100%	−90.66
200 m	100%	−103	100%	−92.42	100%	−94.96
250 m	100%	−104.54	100%	−106.66	98%	−105.74
300 m	100%	−113.38	100%	−111.18	100%	−106.54
350 m	100%	−113.96	100%	−115.88	98%	−114.72
400 m	100%	−117.48	96%	−112.88	96%	−121.16
450 m	100%	−118.2	100%	−117.72	100%	111.42
500 m	100%	−114.24	100%	−115.18	100%	112.32
550 m	100%	−118.46	100%	−114.8	100%	−111.52
600 m	100%	−116.86	100%	−124.28	100%	−117.82

**Table 7:** Reliability results for different distances and packet sizes with the antenna on the 1st, 2nd, and 3rd floor.

P-Size	P-Size: 16 Bytes						P-Size: 32 Bytes						P-Size: 64 Bytes					
A-Height	1st Floor		2nd Floor		3rd Floor		1st Floor		2nd Floor		3rd Floor		1st Floor		2nd Floor		3rd Floor	
Distance	PDR %	RSSI	PDR %	RSSI	PDR %	RSSI	PDR %	RSSI	PDR %	RSSI	PDR %	RSSI	PDR %	RSSI	PDR %	RSSI	PDR %	RSSI
0 m	100%	−72.3	100%	−53.78	100%	−14.58	98%	−73.24	100%	−36.12	100%	−30.48	100%	−67.2	100%	−29.52	100%	−40.66
50 m	100%	−75.32	100%	−87.20	100%	−79.46	98%	−74.66	100%	−64.72	100%	−81.68	98%	−74.88	100%	−72.16	100%	−85.90
100 m	100%	−89.14	100%	−89.72	100%	−97.16	100%	−85.7	100%	−81.3	100%	−90.3	92%	−83.54	92%	−80.76	100%	−90.44
150 m	100%	−103.58	100%	−115	100%	−91.72	100%	−93.24	100%	−89.36	100%	−104.86	96%	−65.64	98%	−92.16	98%	−97.12
200 m	100%	−109.38	100%	−119.26	100%	−97	100%	−101.34	100%	−90.1	100%	−101.44	100%	−99.58	98%	−94.74	100%	−94.92
250 m	98%	−118.58	100%	−119.46	100%	−107	98%	−112.56	98%	−108.72	100%	−99.54	98%	−103.38	100%	−102.3	100%	−104.32
300 m	100%	−116.76	100%	−119.8	100%	−102.28	98%	−116.8	100%	−106.58	100%	−104.96	98%	−105.26	100%	−104	96%	−102.22
350 m	96%	−124.34	100%	−117.82	100%	−109.24	100%	−113	100%	−105.52	98%	−111.42	90%	−112.8	100%	−106.8	98%	−109.5
400 m	100%	−117.64	98%	−123.86	100%	−114.18	100%	−104.46	100%	−114	100%	−107	96%	−111.98	100%	−103.4	90%	−117.92
450 m	92%	−124	92%	−127.9	96%	−118.5	100%	−108.34	100%	−116.34	100%	−116.84	100%	−110.44	94%	−120.24	94%	−119.22
500 m	100%	−124.28	100%	−123.34	100%	−119.26	100%	−120.72	100%	−118.9	96%	−119.38	92%	−119.36	98%	−113.46	100%	−112.3
550 m	78%	−131.16	86%	−124.28	96%	−124.54	76%	−126.14	94%	−119.76	92%	−124.72	76%	−116.64	80%	−118.22	92%	−126.68
600 m	78%	−131.12	90%	−128	100%	−127.6	98%	−115.36	100%	−121.4	90%	−124.98	98%	−125.2	100%	−112.6	98%	−121

**Table 8:** Latency results for different distances, packet sizes and antenna heights.

Packet Size	16 Bytes						32 Bytes						64 Bytes					
Antenna Height	0 m		1 m		2 m		0 m		1 m		2 m		0 m		1 m		2 m	
Distance	L (MS)	RSSI	L (MS)	RSSI	L (MS)	RSSI	L (MS)	RSSI	L (MS)	RSSI	L (MS)	RSSI	L (MS)	RSSI	L (MS)	RSSI	L (MS)	RSSI
0 m	120	−21.96	16	−41.3	8	−48.16	233	−27.42	220	−24.88	208	−42	760	−35.18	749	−56.6	560	−55
50 m	125	−80.98	84	−99.06	8	−85.86	252	−92.82	240	−78.82	222	−84.00	775	−99.80	760	−76.86	576	−77.10
100 m	133	−92.32	91	−108	5	−86.58	260	99.24	245	−89.88	226	−96.48	779	−104.54	764	−91	593	−86.72
150 m	141	−95.62	101	−109.8	10	−89.8	265	−94.84	255	−86.32	231	−92.8	788	−92.48	768	−93.52	595	−90.66
200 m	146	−106.94	111	−110.42	21	−103	272	−99.98	265	−92.52	240	−92.42	798	−102.00	774	−86.44	604	−94.96
250 m	157	−103.30	118	−112.84	29	−104.54	279	−99.98	270	−97.5	281	−106.66	806	−106.34	776	−104.32	618	−105.74
300 m	167	−108.94	126	−117.28	33	−113.38	287	−111.62	280	−100.66	287	−111.18	812	−121.92	785	−107.9	646	−106.54
350 m	176	−114.86	137	−124.26	50	−113.96	293	−111.64	291	−108.28	290	−115.88	820	−120.20	789	−115.72	655	−114.72
400 m	179	−118.20	142	−127	56	−117.48	306	−114.00	298	−106.68	295	−112.88	830	−127.42	794	−118.14	667	−121.16
450 m	199	−115.94	153	−124.1	60	−118.2	361	−115.60	349	−111.86	324	−117.72	848	−131.00	805	−118.8	695	−111.42
500 m	213	−122.60	164	−128.76	64	−114.24	373	−111.66	352	−111.18	335	−115.18	862	−120.70	800	−127.8	718	−112.32
550 m	218	−120.58	172	−129.12	69	−118.46	394	−113.00	366	−120	352	−114.8	867	−121.50	811	−121.84	726	−111.52
600 m	230	−130.92	186	−130.66	77	−116.86	442	−123.00	420	−128.52	409	−124.28	880	−135.60	817	−128.78	745	−117.82

**Table 9:** Latency results for different distances, packet sizes and antenna heights on different floors of IT Building 3.

Packet Size	16 Bytes						32 Bytes						64 Bytes					
Antenna Height	1st Floor		2nd Floor		3rd Floor		1st Floor		2nd Floor		3rd Floor		1st Floor		2nd Floor		3rd Floor	
Distance	L (MS)	RSSI	L (MS)	RSSI	L (MS)	RSSI	L (MS)	RSSI	L (MS)	RSSI	L (MS)	RSSI	L (MS)	RSSI	L (MS)	RSSI	L (MS)	RSSI
0 m	26	−72.3	24	−53.78	23	−14.58	244	−73.24	236	−36.12	237	−30.48	845	−67.2	771	−29.52	738	−40.66
50 m	33	−75.32	24	−87.20	25	−79.46	258	−74.66	249	−64.72	241	−81.68	858	−74.88	784	−72.16	776	−85.90
100 m	49	−89.14	47	−89.72	51	−97.16	291	−85.7	281	−81.3	260	−90.3	870	−83.54	788	−80.76	786	−90.44
150 m	54	−103.58	49	−115	62	−91.72	332	−93.24	299	−89.36	376	−104.86	898	−65.64	795	−92.16	790	−97.12
200 m	59	−109.38	57	−119.26	60	−97	347	−101.34	303	−90.1	288	−101.44	908	−99.58	798	−94.74	789	−94.92
250 m	66	−118.58	59	−119.46	59	−107	387	−112.56	379	−108.72	362	−99.54	938	−103.38	805	−102.3	795	−104.32
300 m	70	116.76	70	−119.8	65	−102.28	398	−116.8	393	−106.58	365	−104.96	958	−105.26	808	−104	805	−102.22
350 m	77	−124.34	73	−117.82	68	−109.24	425	−113	407	−105.52	371	−111.42	973	−112.8	809	−106.8	806	−109.5
400 m	80	−117.64	79	−123.86	72	−114.18	442	−104.46	417	−114	408	−107	980	−111.98	823	−103.4	817	−117.92
450 m	94	−124	85	−127.9	73	−118.5	451	−108.34	422	−116.34	409	−116.84	990	−110.44	825	−120.24	826	−119.22
500 m	104	−124.28	104	−123.34	94	−119.26	516	−120.72	436	−118.9	409	−119.38	1024	−119.36	831	−113.46	828	−112.3
550 m	152	−131.16	108	−124.28	97	−124.54	519	−126.14	447	−119.76	417	−124.72	1059	−116.64	840	−118.22	829	−126.68
600 m	198	131.12	124	−128	109	−127.6	570	−115.36	470	−121.4	419	−124.98	1102	−125.2	845	−112.6	830	−121

## Data Availability

The original contributions presented in the study are included in the article, further inquiries can be directed to the corresponding authors.
